# Sonocatalytic degradation of bisphenol A in aqueous solution: A review^☆^

**DOI:** 10.1016/j.ultsonch.2026.107745

**Published:** 2026-01-17

**Authors:** Hyunjin Shin, Hak-Hyeon Kim, Sujin An, Narae Yang, Chang Min Park, Min Jang, Byung-Moon Jun, Yeomin Yoon

**Affiliations:** a Department of Environmental Science & Engineering, Ewha Womans University, Seoul 03760, the Republic of Korea; b Department of Environmental Engineering, Kyungpook National University, 80 Daehak-ro, Buk-gu, Daegu 41566, the Republic of Korea; c Department of Environmental Engineering, Kwangwoon University, 20 Kwangwoon-Ro, Nowon-Gu, Seoul 01897, the Republic of Korea; d Department of Environmental Science and Engineering, Kyung Hee University, 1732 Deogyeong-daero, Giheung-gu, Yongin-si, Gyeonggi-do 17104, the Republic of Korea

**Keywords:** Sonodegradation, Sonocatalysis, Bisphenol A, Degradation mechanisms, Water treatment

## Abstract

Owing to the expansion of the manufacturing industry, bisphenol A (BPA) is being discharged into aquatic environments, posing a global concern. Numerous studies have recognized the adverse effects of BPA exposure on ecosystems and humans. Therefore, advanced remediation technologies are gaining increasing attention. Beyond the achievements of conventional water treatment processes, sonocatalysis provides several benefits, including effectiveness under ambient conditions, promising mineralization efficiency, and compatibility with nanostructured or hybrid catalysts. This review presents progress and developments made over the past ten years in the field of sonocatalysis related to BPA. Focusing mainly on BPA sonodegradation mechanisms, the effects of solution chemistry (e.g., pH, temperature, naturally occurring ions, natural organic matter, and scavengers), ultrasonication parameters (e.g., ultrasonic frequency, power, and operation mode), and the physicochemical properties of BPA (e.g., pK_a_, hydrophobicity, and molecular configuration) were evaluated. Overall, sonocatalysis demonstrated competent BPA degradation, whereas hybrid systems (e.g., O_3_, sono-Fenton, and ultraviolet/visible light irradiation) enhanced radical utilization. Finally, we discuss the current limitations and potential areas for future research, with the aim of guiding subsequent investigations towards practical applications.

## 1 Introduction

Bisphenol A (BPA) is used as a monomer for synthesizing polycarbonate plastics [1] and resins [2] that are utilized in manufacturing a variety of goods from common household products (e.g., food containers and bottles for beverages) to electronic devices, food preservatives, dental sealants, and baby products [1]. In North America and Europe, BPA production increased by 37 % between 1996 and 2014 [3], and from 2016 to 2022, the global market experienced a compound annual growth rate of approximately 4.8 % [4]. BPA is primarily discharged from microplastics in municipal and industrial wastewater treatment plants [5] and has been widely detected in aquatic environments in Europe, South America, Asia, and North America [6]. A study on the Xochimilco wetlands of southern Mexico City reported a maximum BPA concentration of 1.4 × 10^5^ μg L^−1^ in surface water [7]. According to an analysis on European seawater, the median concentration of BPA was 22.2 ng L^−1^, whereas higher levels of 4,800 ng L^−1^ were also observed [8]. In the Bohai Bay seawaters of China, the mean BPA concentration was 137 ng L^−1^, suggesting the persistence of BPA despite production and application regulations since 2008 [9]. Owing to its structural similarity to hormones, such as estrogen, testosterone, and aldosterone, BPA can overstimulate their receptors, potentially leading to endocrine dysfunction [10]. In addition, increasing evidence suggests that BPA exposure and bioaccumulation may be linked to genomic instability, carcinogenesis [11], and metabolic disorders [12], thereby raising concerns regarding its impact on human health.

Conventional treatment processes for BPA removal include biodegradation such as with lagoon treatment, aerated filters, and activated sludge [13]; physical treatment methods such as coagulation, sedimentation, and filtration [14]; and chemical approaches such as chlorination [15]. However, conventional treatment methods require prolonged reaction times and are less efficient [16], particularly for trace concentrations of BPA [14]. Consequently, recent research has shifted towards advanced technologies, such as membrane filtration [17], adsorption [18], membrane bioreactors [19], and advanced oxidation processes (AOPs) [20], [21], which show relatively high BPA removal efficiencies.

Various AOPs have been employed for BPA degradation, including ultraviolet (UV) irradiation, O_3_/H_2_O_2_ photocatalysis, and ultrasonication [22], [23], [24]. Among these, ultrasonication is considered promising because it is time-efficient, simple, and operable at ambient temperatures and pressures with minimal generation of secondary pollutants, such as sludge or concentrated wastewater [25], [26]. Ultrasound waves induce cavitation through rapid pressure fluctuations in the bulk solution, where bubbles repeatedly form, grow, and collapse [27]. The collapse of these bubbles under extreme local temperatures and pressures generates ^•^OH, a reactive species capable of degrading chemicals within the bubble, at the interface between the bubble and solution, and in the bulk solution [28]. This process is competent in degrading compounds with hydrophobic properties such as BPA [29]. However, the short lifespan and high reactivity of ^•^OH often lead to reactions with competing species or recombination. This reduces the efficiency of sonication and consequently results in high operating costs, particularly for on-site applications [25], [30].

To overcome these limitations, various catalysts and ultrasonication-based hybrid systems have been developed to remove BPA [31]. A number of catalysts such as copper film (CF) [23], MXenes [32], biochar (BC) [33], reduced graphene oxide [34], carbon nanofibers (CNFs) [35], graphitic carbon nitride (GCN) [36], and carbon nanotubes [37] have been employed in conjunction with oxidants such as H_2_O_2_ [38], peroxydisulfate (PDS) [39], and peroxymonosulfate (PMS) [34] in an attempt to improve BPA sonodegradation performances. Metal oxides such as BaTiO_3_ [40] and ZnO [41] have demonstrated significant enhancement in their piezocatalytic effects when combined with ultrasound [42]. Furthermore, recent studies have incorporated ultrasonication hybrid systems that feature UV irradiation [43], ozone [44], and photo/Fenton processes [45] to achieve synergistic effects.

Since 2017, the literature on the sonocatalytic degradation of BPA has increasingly focused on nanosized catalysts, the activation of PMS by ultrasonication, and the integration of sonodegradation with photocatalysis [46]. However, reviews on the degradation of BPA are limited, and focused analyses that specifically address the sonocatalytic degradation of this organic compound remain relatively underexplored. Therefore, this review aims to provide a thorough insight into the development of BPA sonodegradation over the past decade. The primary objectives were to (i) investigate the influence of different water quality conditions, physicochemical properties of BPA, and sonication parameters on removal efficiency; (ii) evaluate the performance of catalyst-assisted and ultrasonication hybrid systems; and (iii) elucidate the possible mineralization pathways of BPA. Additionally, a brief overview of upcoming research directions is provided to address current knowledge gaps.

## 2 Ultrasonication treatment of BPA

### 2.1 Degradation affected by water quality and sonication conditions

#### 2.1.1 pH

Maintaining an optimal pH is critical for the sonodegradation of BPA because it affects ^•^OH formation efficiency and modifies the hydrophilic–hydrophobic balance of the compound, thereby altering its affinity for the bubble interface [21]. Different pH conditions were employed once 500 mg L^−1^ GCN was added to a 10 mg L^−1^ BPA solution under ultrasonic conditions of 35 kHz and 50 W [36]. The rate constants at pH 2 and pH 4–8 were 10.0 × 10^-3^ min^−1^ and approximately 9.0 × 10^-3^ min^−1^, respectively. However, a rapid decrease to 6.0 × 10^-3^ min^−1^ occurred at pH 10, which was ascribed to the ionization of BPA into bis-phenolate owing to its ionization constants of 8–9.6 [47]. This was inferred to increase the electrostatic repulsion and weaken the hydrophobic reactions with GCN [48]. Corresponding results were obtained in a high frequency hydrodynamic acoustic cavitation procedure with 291 μM PDS and 89 μM Fe^2+^ [49]. An increase in pH from 3.5 to 11.0 was related to a decrease in degradation rate, likely due to the repulsion between BPA anions and negatively charged bubbles, where oxidative radicals were predominantly generated. Moreover, pH levels above 3.5 might have restricted the ability of Fe^2+^ to activate PDS [50].

CoFe_2_O_4_-GO (CoFeO-GO) and CoFe_2_O_4_-g-C_3_N_4_ (CoFeO-GCN) were fabricated using ultrasonication-assisted coprecipitation [39]. BPA degradation efficiency was investigated in aqueous solutions ([BPA] = 12.5 mg L^−1^, [PDS] = 2 mM, and catalyst dose 100 mg L^−1^) with varying pH levels. At pH 6.8, the highest degradation rate of approximately 90 % was achieved owing to the π-π forces between the BPA molecules and catalysts at their neutral forms. However, less than 50 % of BPA was degraded at pH 7.8. As point of zero charge of Co-FeO-GO and CoFeO-GCN (pH_pzc_; approximately 6.5 and 6.0, respectively) is lower than pH 7.8, the catalysts featured a negatively charged surface [51], [52], causing the electrostatic repulsion between the catalyst and partially ionized BPA. A pH level of 3.8 provided slightly better performance, as the attraction between PDS and positively charged catalysts prompted the generation of SO_4_^•-^ [39].

A research on ultrasonication process combined 0.1 g L^−1^ PMS with Bio-MOF-1 (BMOF) [53]. Adenine linkers were incorporated into a metal–organic framework to synthesize the BMOF composite. The maximum degradation efficiency at pH 5.0 and pH 7.0 was attributed to the enhanced activation capacity of PMS and the ionization of BMOF. At pH levels lower than 9.0, PMS was likely converted to HSO_5_^-^, thereby improving radical production. When solution pH was lower than pH_pzc_ (8.43), the positively charged BMOF surface was presumed to mediate PMS adsorption [54]. At pH 3.0 however, the removal rate was significantly inhibited. The peroxide bonds of PMS were suspected to form hydrogen bonds with H^+^, deactivating PMS and suppressing radical formation [55]. At pH 9.0, which was close to the pK_a_ of PMS, dissociation state of PMS had occurred, adversely affecting PMS activation and the degradation rate of BPA. In a separate study, PDS was profoundly activated in a basic solution by the addition of NaOH [23]. Owing to enhanced ^•^OH production rate, the degradation rate of BPA increased from 82.6 % at pH 3 to 90.3 % at pH 11 [56]. Consistently, SO_4_^•-^ was converted into ^•^OH at pH 8, increasing the reaction rate compared to that at pH 6 [20].

In another study, pH variations had minimal effect on the sonolysis of 0.5 mg L^−1^ BPA in ultra-pure water under ultrasonic conditions of 20 kHz and 71 W L^−1^; the kinetic constants at pH 3, 6.6, and 9 were 0.030, 0.024, and 0.028 min^−1^, respectively [57]. However, the extent of the increase in the removal rates differed by pH considerably following the addition of 500 mg L^−1^ Pd/CeO_2_. Although the rate constant increased marginally to 0.031 min^−1^ at pH 3, significant increases of 0.052 and 0.049 min^−1^ were observed at pH 6.6 and 9, respectively [57].

#### 2.1.2 Temperature

In general, elevated temperatures are favorable for the sonodegradation of contaminants [31]. The efficiency of ^•^OH generation improves because of the lower cavitation threshold and increased vapor pressure [58], whereas the reduced contaminant solubility enhances migration towards the liquid–gas interface [59]. However, vapor-filled cavities tend to collapse less violently than gaseous cavities, which potentially weakens radical formation [60]. Thus, the ideal temperature should be selected by considering the physicochemical properties of target molecules and their reaction kinetics with radicals [61].

In a sono-Fenton hybrid experiment conducted under 20 kHz and 40 W ultrasound with 15 mg L^−1^ BPA solution, the BPA degradation efficiency gradually increased with temperature (21.8 % at 298 K to 25.9 % at 313 K) [62]. However, despite the larger quantities of bubbles formed at higher temperatures, they exhibited less effective cavitation collapse [63]. These bubbles hinder ultrasonic transmission and mitigate the effective use of energy derived from ultrasonication. Therefore, the enhancement of BPA degradation performance was primarily attributed to the temperature-accelerated Fenton reaction. In agreement with this, an optimum sonication temperature of 293 K [64] and maximum BPA removal at 313 K [65] have been suggested in other studies. Darsinou et al. observed an overall negative effect of increased temperatures in a sono-activated persulfate oxidation system [66]. Under 20 W L^−1^ and 20 kHz sonication, the BPA degradation efficiency decreased from 90 % at 30 °C to 25 % at 60 °C. Although thermolysis of the applied sodium persulfate was assumed to have improved [67], the relatively high temperature caused several detrimental effects. Degassing of the solution could decrease the quantity of gas nuclei [68], and an increased vapor content could lead to mild bubble implosion [69]. A similar result was reported for the sonodegradation of estrogens [70].

The kinetic constants of BPA degradation increased nearly three-fold from 0.07 min^−1^ at 283 K to 0.18 min^−1^ at 313 K [71]. This was ascribed to the exponential increase in both radical production speed and Fe^2+^/Fe^3+^ redox cycling rates [72]. Based on the Arrhenius equation, activation energies for the second stage of reaction of catalyst/H_2_O_2_ and catalyst/ultrasonication /H_2_O_2_ systems were calculated as 31.2 kJ mol^−1^ and 23.4 kJ mol^−1^, respectively, revealing a significant reduction with the application of ultrasonication. Both values exceeded those typically observed for diffusion-controlled reactions (10–13 kJ mol^−1^) [73]. Therefore, it was apparent that intrinsic chemical reactions, rather than mass transfer, governed the observed kinetics [73]. In a separated study, the efficiency of BPA degradation was assessed under 35 kHz and 50 W ultrasonication, combined with mechanical stirring at 300 rpm [63]. The highest kinetic constant was obtained at 20 °C (7.6 × 10^-3^ min^−1^), followed by 4.0 × 10^-3^ min^−1^ at 30 °C and 2.5 × 10^-3^ min^−1^ at 10 °C. At 30 °C, elevated vapor pressure inside bubbles may have suppressed the pressure and intensity of heat caused by bubble collapse, leading to decreased reaction speed [74]. Comparable inhibitory effects on rapid gas formation have been observed during sonodegradation of dyes [75].

In contrast, a peak degradation efficiency of approximately 90 % was achieved at 40 °C when 400 kHz ultrasound was applied to a 0.01 mM BPA solution [29]. Consistent with the previous literature [76], the lowered cavitation thresholds could possibly contribute to the efficiency of the system by promoting the cavitation phenomena. However, removal rate decreased to 80 % at 60 °C, likely because of a decrease in solution surface tension, which may have limited the overall sonochemical performance [29].

#### 2.1.3 Background common ions and natural organic matter

Background ions and natural organic matter are generally regarded as radical scavengers that can negatively affect AOP degradation efficiency [77]. However, anions such as Cl^-^ [78] and organic surfactants, including sodium dodecylbenzene sulfonate and cetrimonium bromide [53] are known to promote BPA removal under certain conditions. They may also serve as indicators of the presence or involvement of reactive species [79].

Hematite nanoparticles were synthesized by precipitating FeCl_3_·H_2_O and urea using ammonium hydroxide [80]. In conjunction with PDS, the resulting sonocatalyst achieved 98.6 % BPA removal. Once various anions were present, the removal rates decreased to 83.6 % (Cl^-^), 61.6 % (SO_4_^2-^), and 51.8 % (HPO_4_^2-^). This was attributed to the relatively low oxidation potentials of Cl^•^ and Cl_2_^•-^, which were formed by the interaction between Cl^-^ and SO_4_^•-^ [77]. Moreover, HPO_4_^2-^ could produce hydrogen phosphate radicals with significant inhibitory effects, in addition to occupying the catalyst surface and serving as a complexing agent for iron [81].

PDS was used with CF consisting of 0.20 % copper and a polyethylene base, achieving 99.5 % initial BPA degradation [23]. Consequently, the effects of various ions and natural organic matter on BPA degradation were evaluated. NO_3_^–^ lowered the degradation rate from 99.5 % to 96.6 %, presumably by reacting with sulfate radicals to form NO_3_^•^ [82]. In contrast, degradation rates were improved to nearly 100 % in the presence of Cl^-^, which was attributed to the reaction between copper ions and PDS facilitated by the formation of cuprous complexes [83]. The addition of *tert*-butanol and methanol hindered BPA degradation to 96.8 % and 96.0 %, respectively, suggesting the involvement of both ^•^OH and SO_4_^•-^ in the reaction. Notably, *tert*-butanol reacts with ^•^OH at a much faster rate (k = 3.8–7.6 × 10^8^ M^−1^ s^−1^) than with SO_4_^•-^ (k = 4.0–9.1 × 10^5^M^−1^ s^−1^), whereas methanol shows a smaller rate disparity (approximately one order of magnitude) between the two radicals [84]. Therefore, SO_4_^•-^ was assumed to play a dominant role, similar to results reported in another study [85].

Sonodegradation experiments of BPA and methylene blue (MB) were conducted utilizing municipal landfill leachate at pH 8.3 [86]. Both pollutants exhibited significant reductions in their removal rates, from over 99 % to 10 % for BPA and from 89.8 % to 9.39 % for MB. Owing to the presence of additional contaminants, this decrease may be due to competition for free radicals [87]. However, when the catalyst dose and persulfate concentration were increased, degradation rates of BPA and MB recovered to 88.8 % and 93.9 %, respectively. Leachate compounds were assumed to enhance degradation through interacting with contaminants via π-π bonding, chelation, and ion exchange [88]. Although humic acid is generally recognized as a radical scavenger, in this case, certain phenolic and quinone components consisting humic acid were found to induce the formation of high-energy radicals via PMS decomposition [89]. In a separate study, the addition of 10 mM HCO_3_^–^ reduced the BPA degradation efficiency from 98.1 % to 59.5 % [85]. This was associated with the production of CO_3_^•-^ and HCO_3_^•^ which have lower redox potentials than those of SO_4_^•-^ and ^•^OH [90]. The addition of HCO_3_^–^ also created presumably unfavorable alkaline conditions [56]. Furthermore, the reaction between HCO_3_^–^ and Fe^2+^ on the modified sludge biochar catalyst may have formed compounds that block active sites, such as siderite or Fe^2+^/Fe^3+^ (oxy)-hydroxyl carbonate [91].

#### 2.1.4 Promoters and scavengers

PDS exhibited a higher promoter activity than H_2_O_2_ for BPA degradation, achieving 100 % and 3.37 % degradation, respectively [23]. 100 mM of each substance was employed to 0.1 mM BPA solution with ultrasonic parameters of 37 kHz, 97.17 W, and 60 °C. The relatively competent degradation ability of PDS could be attributed to its thermal activation and generation of SO_4_^•-^ [92], [93]; a substantial increase from < 1 % to nearly 100 % removal was observed in relation to temperature increase from 30 °C to 70 °C. Additionally, the longer lifetime and greater redox potential of SO_4_^•-^ may have contributed to these findings, similar to those of a degradation study on ibuprofen [94]. The quenching experiments utilizing 2.0 mM furfuryl alcohol and beta-carotene as scavengers revealed the dominant role of ^1^O_2_ in the BMOF/PMS/ ultrasonication procedure [53]. The high degradation efficiencies of BPA and carbamazepine contrasted with the limited removal rate of atrazine, suggesting that ^1^O_2_ could attack the electron-dense regions of BPA [95].

Jun et al. observed persulfate activation by ultrasound irradiation under 20 kHz (33.6 ± 4.3 W), 28 kHz (33.3 ± 6.2 W), and 300 kHz (37.6 ± 1.4 W) [96]. In addition, various temperature ranges from 5 to 10 ℃ to 55–60 ℃ were applied. For temperatures below 55 ℃, rate constants ranged from 0.01 to 0.05 min^−1^ for both ultrasonication alone and ultrasonication with persulfate, indicating only limited synergy. In contrast, for ultrasonication with persulfate at 55–60 ℃, SO_4_^2-^ concentrations significantly increased to approximately 20–30 mg L^−1^, and the rate constant for 20 kHz increased to approximately 0.11 min^−1^. The enhanced persulfate activation may be attributed either to local temperature increases induced by intense cavitation at 20 kHz [97] or to elevated bulk temperature itself, therefore requiring further investigation [96].

CuS/BaWO_4_ catalysts with varying molar ratios were synthesized by reacting sodium tungstate with barium chloride, followed by grinding and supplementation with copper nitrate and sodium thiosulfate [98]. While 80 % CuS composition demonstrated 71.0 % ± 1.46 % BPA degradation efficiency, further addition of 0.5 μmol K_2_S_2_O_8_ increased the removal rate to 95.2 % ± 0.21 % and accelerated the reaction. Implementation of D-Mannitol, edetate disodium, and ascorbic acid reduced the removal rates to 15.3 % ± 0.98 %, 9.64 % ± 1.47 %, and 1.36 % ± 1.16 %, respectively, indicating significant roles of ^•^OH and h^+^. The minor effect of N_2_ reflected minimal O_2_^•-^ involvement [98]. A separate study demonstrated that the addition of solid particles increased BPA removal by approximately 10 % [44]. The positive outcomes of the sand and glass particles were attributed to enhanced oxidant production. This was achieved by reducing cavitation thresholds, elevated solution temperature, and improved hydrodynamic effects [58]. Moreover, the heterogeneous surface could provide additional cavitation nuclei [99]. Presumably, the irregular configuration of glass particles accounted for slightly higher removal rates than those of sand [44].

The dominant role of ^•^OH in the ultrasonication/schwertmannite/H_2_O_2_ system was elucidated through quenching experiments using n-butanol, isopropanol, and benzoquinone [71]. Isopropanol, a strong quencher of free ^•^OH, reduced the degradation efficiency from 98.0 % to 12.1 %. A more pronounced reduction to 5.2 % was observed with n-butanol, suggesting the active involvement of surface-bound ^•^OH on the catalyst [100]. In contrast, the scavenging of HO_2_^•^ and O_2_^•-^ by benzoquinone [72] caused only a slight decrease in degradation efficiency [71]. The co-precipitation of rice bran with ferric chloride hexahydrate and rice husk with iron(II) chloride yielded two distinct BC composites [23]. The catalyst used in the sono-Fenton experiments achieved approximately 94 % degradation. However, the removal rates were notably hindered by ethanol (by approximately 10 %), and ions such as SO_4_^2-^, CO_3_^2–^, and Cl^-^ reduced the removal efficiency to approximately 30 %, 10 %, and 50 %, respectively. These effects were ascribed to ^•^OH scavenging and the generation of radicals with relatively weak oxidation potentials [101].

#### 2.1.5 Ultrasonication frequency, power, and reactor type

Heterostructured β-Bi_2_O_3_/Bi_2_O_2_CO_3_ catalysts were synthesized through calcination at high temperature via thermal annealing and demonstrated near complete BPA degradation under continuous ultrasonication at 35 and 72 kHz, respectively [102]. When a strong microjet collapsed at low frequencies, BPA could presumably diffuse towards the gaseous zone, undergoing oxidation and pyrolysis [103]. However, increased frequencies of 100 and 170 kHz exhibited approximately 70 % and 40 % degradation, respectively; results contrary to those of previous studies on phenol and 4-chlorophenol [104]. Utilizing sugarcane juice as an eco-friendly reducing agent and stabilizer, α-MnO_2_ nanorods were synthesized [86]. A slight decrease in BPA removal rate in continuous mode was observed with increasing frequency, from approximately 90 % at 37 kHz to 85 % at 80 kHz. This may be attributed to reduced intervals between the compression and rarefaction phases, leading to a diminished cavitation intensity and a smaller bubble resonance radius [105]. Similarly, Sunasee et al. suggested an increase in the bubble size at relatively lower frequencies [63]. At a frequency of 35 kHz, mechanical stirring at 300 rpm could divide larger bubbles into additional bubbles with smaller diameters, thus improving ^•^OH production [106]. However, ^•^OH formation has also been reported to increase with high-frequency ultrasonication [107]. The intensities of 7-hydroxycoumarin were approximately 50, 180, and 400 relative fluorescence units at 20, 200, and 400 kHz, respectively, indicating an enhanced ^•^OH production capacity [107].

Fletcher et al. reported a 94.2 ± 0.7 % BPA degradation rate and an 86.2 ± 3.3 % degradation efficiency with a dual-frequency operation system of 37/20 kHz and 80/20 kHz, respectively [108]. The initial reaction rate under 37/20 kHz was approximately threefold higher than that of the 80/20 kHz system, while applied ultrasonic power under continuous mode was 272 ± 3.92 and 210 ± 6.94 W L^−1^, respectively. The frequency disparity was proposed to create asynchronous bubble oscillations, thereby suppressing coalescence and increasing availability of active cavitation bubbles [109]. Additionally, dual-frequency operation was associated with elevated acoustic pressure and increased vapor accumulation inside the bubble, consistent with predictions from cavitation dynamics modelling [110].

The removal efficiency of contaminants was positively correlated with the intensity of ultrasonication in continuous mode [43]. As the intensity increased, the half-life of BPA decreased from 13 to 2 min and that of pyrene decreased from 39 to 3 min. Corresponding rate constants for BPA degradation at intensities of 9, 17, 26, and 43 W cm^−2^ were 0.05, 0.11, 0.21, and 0.32 min^−1^, respectively, whereas for pyrene the values were 0.02, 0.04, 0.05, and 0.21 min^−1^, respectively. At 43 W cm^−2^, BPA reached a maximum degradation efficiency of 92 %, whereas pyrene was nearly completely removed [43]. Under 24 kHz ultrasound, BPA degradation increased from 38 % at an amplitude of 25  μm to 61 % at 125  μm [111]. This enhancement was attributed to the increased amplitude, which promoted greater ^•^OH production, indicating the efficiency of the process at low frequencies [112]. Comparable results were observed in a separate study [23], in which turbulent mixing caused by high-intensity ultrasonication induced larger quantities of cavitation bubbles [113].

Different results have been reported in a separate study on continuous ultrasound irradiation [102]. Increasing the intensity from 0.066 to 0.11 W cm^−2^ significantly increased the removal rates from approximately 30 % to 70 %, which was possibly due to enhanced formation of free radical species [61]. However, for intensities above 0.11 W cm^−2^, rather consistent degradation efficiencies in the range of 70 % to 80 % were observed [102]. Similarly, the removal rate of BPA increased significantly from 69.6 % at 100 W to 97.6 % at 250 W, but exhibited a marginal increase to 98.0 % at 300 W [80]. These results suggest that an optimal power density leads to a greater number of bubbles, thereby producing more reactive species. However, the minimal increase in the removal rates at power levels exceeding 250 W suggests a possible disturbance of bubble collapse and sound wave instability [114].

Ultrasonication in pulse mode demonstrated higher degradation efficiency than that in continuous operation [43]. During silent intervals, bubbles could effectively cavitate, progressing into the subsequent active phases and allowing for the diffusion of BPA and pyrene towards the bubbles [115]. Under optimized conditions of 4 s on and 2 s off, removal rates reached 93 % for BPA and nearly 100 % for pyrene [43].

### 2.2 Degradation affected by various catalysts

#### 2.2.1 Non-carbon material-based catalysts

Catalysts appropriate for sonocatalysis are characterized by large surface areas which provide active sites for bubble formation and catalytic activity [116]. They are also chemically and physically stable and exhibit high reusability [117]. Metal oxides have attracted growing attention as non-carbon-based catalysts, which show piezocatalytic effects [42] and are frequently coupled with UV light to demonstrate photocatalytic abilities [118] or engineered to lower their bandgap, allowing activation under visible light range or by sonoluminescence [119]. Meanwhile, carbon-based catalysts possess electrical conductivity, unique porous structures, and relatively low toxicity [120]. In particular, contaminants can undergo hydrophobic π-π interactions, hydrogen bond-mediated adsorption, and electrostatic interactions at active sites of carbon-based catalysts [121].

**Fig. 1:**
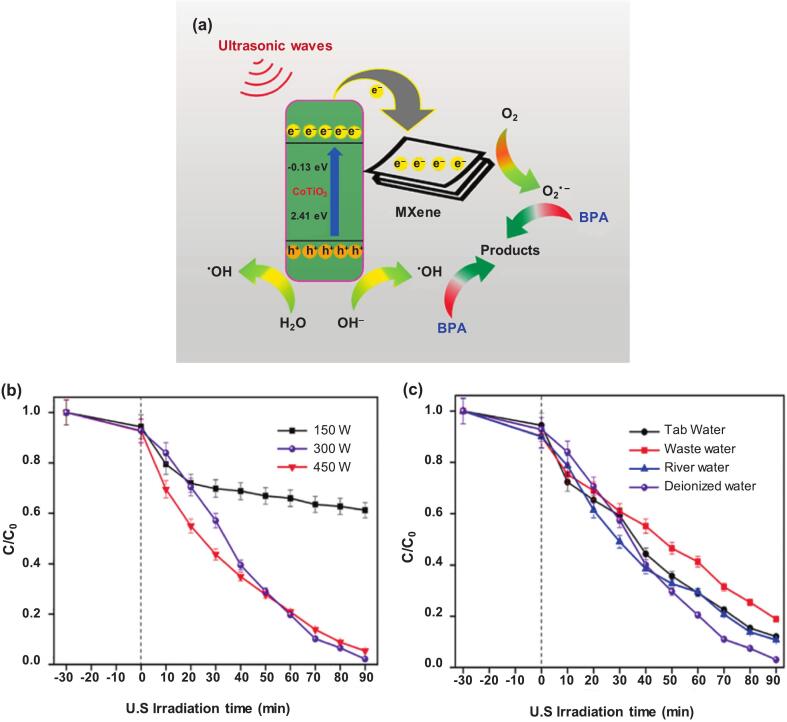
(a) Possible sonocatalytic mechanism of CoT@MX (1:0.5) system in BPA degradation under US irradiation. Sonocatalytic degradation of BPA in (b) various US power and (c) different water matrix. Reaction conditions: catalyst = 0.500 g L^−1^, BPA = 10 mg L^−1^, pH = 5.3, US power = 300 W, and temperature = 25 °C [32].

Various catalysts comprising metals [38], [83], amorphous or crystalline carbon materials [34], [36], photocatalytic semiconductors [122], [123], and their composites [35], [37] have demonstrated efficacy in the removal of organic contaminants such as BPA. A novel CoTiO_3_@MXene (CoT@MX) catalyst was integrated using a liquid self-assembly strategy [32]. The composite material exhibited an increase in Brunauer–Emmett–Teller specific surface area (CoTiO_3_: 8.54 m^2^ g^−1^, MXene: 9.89 m^2^ g^−1^, and CoT@MX: 13.0 m^2^ g^−1^), providing more sites for the adsorption and degradation of BPA. The estimated band gaps were 2.54 eV for CoTiO_3_ and 2.49 eV for MXene, and the heterogeneous interface facilitated the rapid transfer of electrons from CoTiO_3_ to MXene. The reaction mechanism is illustrated in Fig. 1a. The reaction among H_2_O, OH^–^, and h^+^ was proposed to generate ^•^OH, whereas O_2_^•-^, a strong anionic radical, likely arised from the reaction between adsorbed oxygen and e^-^. As shown in Fig. 1b**, a** substantial improvement in BPA degradation was observed (38.7 % and 96.6 % removal at 150 and 300 W, respectively) at a relatively high ultrasonication power, owing to an increase in ^•^OH concentration [124]. However, an intensity of 450 W reduced the removal rate by 2.5 % because the enlarged bubbles could likely impede acoustic wave transmission. Additionally, Fig. 1c shows a significant decline in the removal efficiency for river water (89.9 %), tap water (87.3 %), and wastewater (81.1 %) compared with that for deionized water (96.6 %) [32]. This reduction is likely attributable to the presence of background inorganic and organic compounds in the river, tap water, and wastewater.

**Fig. 2:**
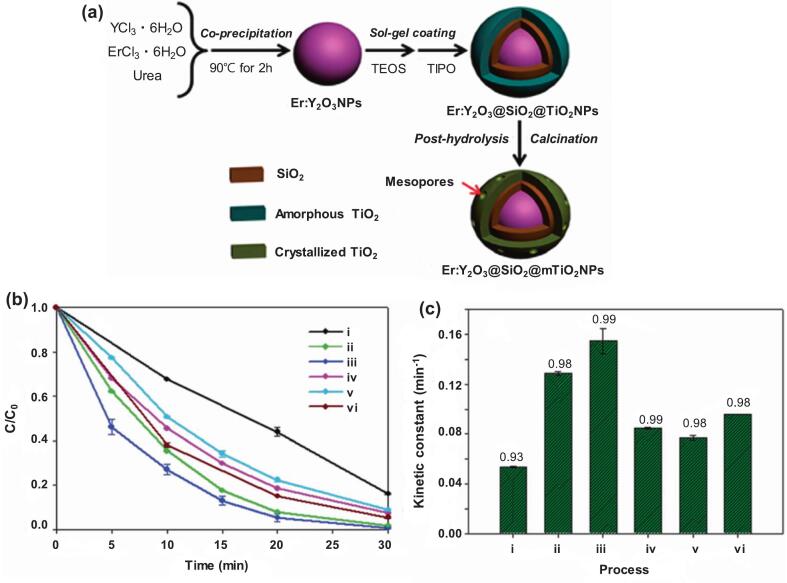
Schematic illustration for combining the UC agent with mesoporous TiO_2_ (a); TIPO = titanium (IV) isopropoxide, TEOS = tetraethyl orthosilicate. The sonocatalytic degradation performance (b) and pseudo first-order kinetic constant (c) of BPA with varied sonocatalysts; (i) US alone; (ii) Er: YO-1@SiO_2_@mTiO; (iii) Er: YO-2@SiO_2_@mTiO; (iv) Er: YO-3@SiO_2_@mTiO; (v) hollow structured mesoporous TiO_2_; and (vi) nonporous Er: YO-2@SiO_2_@TiO composites. In (c), the value above the vertical bar is the coefficient of determination, which is defined as the ratio of the explained variation to the total variation [125].

The preparation of a mesoporous up conversion sonophotocatalyst (Er:Y_2_O_3_@SiO_2_@mTiO_2_) is illustrated in Fig. 2a [125]. First, erbium-doped yttrium oxide nanoparticles were formed via coprecipitation. Subsequently, a silica layer was assembled on the nanoparticles using the Stöber sol–gel method, which was coated with a titania shell. Finally, post-hydrolysis and calcination were performed on the amorphous TiO_2_ shell to create a mesoporous crystalline structure. An ultrasonic system of 300 kHz frequency and 100 W power was applied to a 5 mg L^−1^ BPA solution along with 500 mg L^−1^ of the catalyst. All the obtained nanocomposites appeared to enhance the degradation efficiency of BPA, as shown in Fig. 2b and c. Ultrasonication alone achieved nearly 80 % degradation, whereas the implementation of catalysts demonstrated nearly complete removal within 30 min. Furthermore, assuming pseudo-first-order reactions, the kinetic constants varied significantly depending on the catalyst type. Compared to the rate constant of 0.054 min^−1^ (ultrasonication alone), slightly higher rates of 0.077 and 0.096 min^−1^ were observed when hollow-structured mesoporous TiO_2_ and nonporous Er: Y_2_O_3_-2@SiO_2_@TiO_2_ (Er^3+^: Y_2_O_3_ 3 wt%) were present, respectively. Moreover, mesoporous Er: Y_2_O_3_-2@SiO_2_@mTiO_2_ (Er^3+^: Y_2_O_3_ 3 wt%) maximized the speed of degradation to 0.155 min^−1^, as surface mesopores on the catalyst accelerated cavitation nucleus formation [126], [127]. Moreover, the upconversion agent enabled TiO_2_ activation under visible light by converting incident photons to UV-range photons, generating higher concentrations of oxidative radicals and contributing to overall contaminant degradation [128], [129]. Increasing the proportion of Er^3+^ was preferable to a certain degree. However, at 5 wt%, the rate constant was reduced to approximately 50 % of that at 3 wt% because of the diminished upconversion emission intensity [125].

**Fig. 3:**
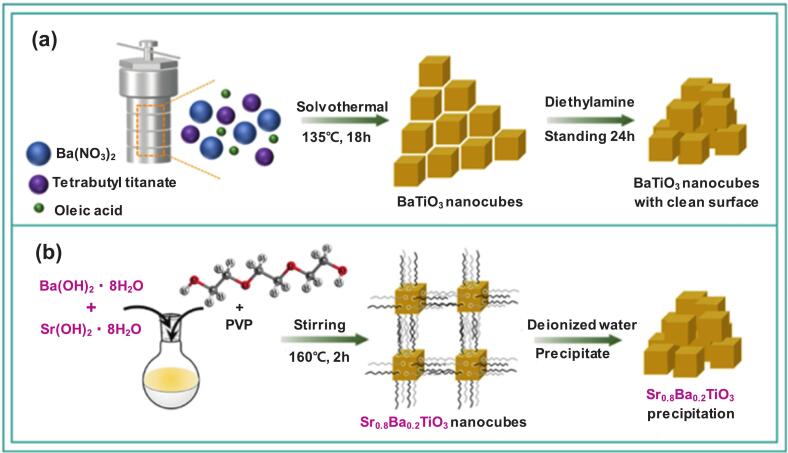
Synthesis scheme of (a) BaTiO_3_ and (b) SrBaTiO solid solution nanocubes; PVP = polyvinyl pyrrolidone [130].

Long et al. compared the BPA degradation performance of BaTiO_3_ and SrBaTiO nanocubes [Sr_x_Ba_1-x_TiO_3_ (0.7 ≤ x ≤ 1.0)] [130]. A solvothermal reaction followed by diethylamine treatment produced BaTiO_3_ nanocubes with clean surfaces (Fig. 3a). In a separate synthesis, SrBaTiO was obtained using a brief sol-precipitation method that involved the dispersion, gradual heating, and stirring of tetrabutyl titanate, strontium hydroxide octahydrate, barium hydroxide octahydrate, ammonia, and polyvinyl pyrrolidone (Fig. 3b). Among the tested catalysts, Sr_0.8_Ba_0.2_TiO_3_ demonstrated the highest BPA removal efficiency of nearly 100 %, followed by BaTiO_3_ and Sr_0.9_Ba_0.1_TiO_3_ each achieving approximately 90 % BPA removal. In terms of pseudo-first-order kinetic constants, the rate constant of Sr_0.8_Ba_0.2_TiO_3_ (0.239 min^−1^) was approximately 1.5-fold that of BaTiO_3_. The favorable outcomes of Sr_0.8_Ba_0.2_TiO_3_ were attributed to the enhanced piezoelectric effect caused by the Ti-O octahedral lattice distortion and lattice asymmetry, combined with the persistent compressive stress induced by sonication. Considering that removal rates declined from approximately 100 % and 90 % to 80 % as ultrasonication power decreased from 300 W and 240 W to 180 W, respectively, higher ultrasonic intensities increased the degradation efficiency. Additional experiments using Sr_0.8_Ba_0.2_TiO_3_ demonstrated removal efficiencies of 99 % for rhodamine B, 67 % for 2,4-dichlorophenol, and 63 % for phenol. The comparatively lower degradation of phenolic compounds was attributed to the structural stability of phenol molecules. [130].

**Fig. 4:**
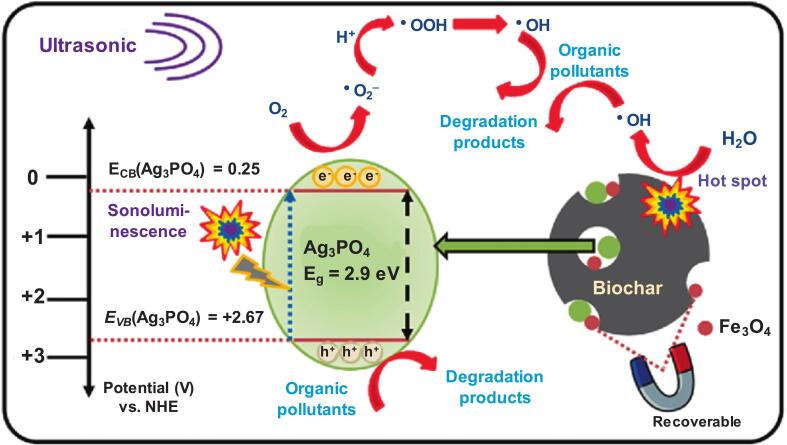
Plausible mechanism of sonocatalytic degradation of organic contaminants in US + Ag_3_PO_4_-Fe_3_O_4_@AB process [131].

The pre-assembled Fe_3_O_4_@AB composites were sonicated in ethanol and introduced into silver nitrate and disodium hydrogen phosphate dodecahydrate solutions, resulting in the formation of Ag_3_PO_4_-Fe_3_O_4_@AB [131]. In ultrasonication experiments, Ag_3_PO_4_-Fe_3_O_4_@AB achieved approximately 80 % and 100 % degradation of BPA and rhodamine B, respectively, which was significantly higher than those of BC (60 %–75 %), activated BC (50 %–65 %), and others (35 %–55 %; Fe_3_O_4_@AB, Fe_3_O_4_, or no catalyst). The doping of Ag_3_PO_4_ onto Fe_3_O_4_@AB was conducive to reinforcing ^•^OH generation, and this increased ^•^OH generation was inferred to be the prime reason for the improved degradation. Two main ^•^OH production mechanisms are proposed in Fig. 4; (i) pyrolysis of H_2_O near cavitation hotspots on Fe_3_O_4_@AB [132] and (ii) sonoluminescence-directed generation of O_2_^•-^ followed by its transition into ^•^OH [133]. Produced from these reactions, ^•^OH and h^+^ were presumably the main reactive species. According to quenching experiments, the rate constants were approximately 0.035 and 0.025 min^−1^ for BPA and rhodamine B, respectively, but decreased to less than 0.005 min^−1^ for both chemicals in the presence of ^•^OH scavengers (inorganic: SO_4_^2-^, CO_3_^2–^, and Cl^-^; organic: methanol, *tert*-butanol, and Suwannee River natural organic matter). Additionally, the inorganic quenchers contributed to h^+^ scavenging by interacting with the holes to create SO_4_^•-^, CO_3_^•-^, and Cl^•^. Finally, the removal rates for orange G and 2,4-dichlorophenol were approximately 60 % and 90 %, whereas rate constants were 0.013 and 0.038 min^−1^, respectively, suggesting the wide applicability of Ag_3_PO_4_-Fe_3_O_4_@AB [131].

**Fig. 5:**
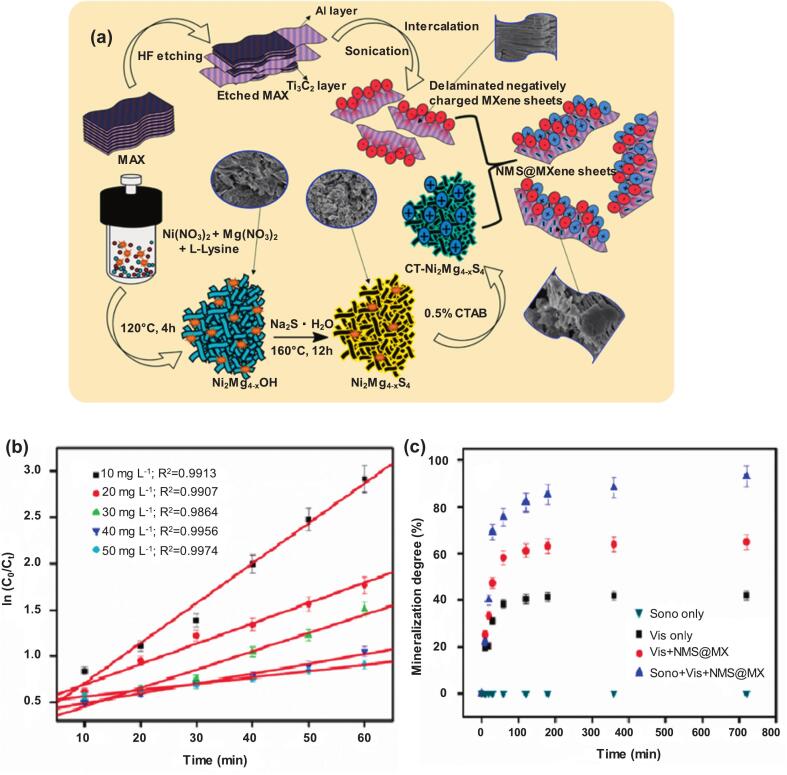
(a) Schematic illustration of the synthesis of NMS@MX sonophotocatalyst. Effect of initial BPA concentration on its (b) degradation and (c) mineralization by the Sono + Visible light (Vis) + NMS@MX system; CTAB = cetrimonium bromide aqueous solution (experimental conditions: NMS@MX 1 g L^−1^, 970 kHz frequency, 400 W US power, and 0.180 mW cm^−2^ light intensity) [24].

Fig. 5a shows the hydrothermal synthesis of nickel–magnesium sulfide Ti_2_C_2_T_x_ MXene (NMS@MX), which produced a Z-scheme heterojunction sonophotocatalyst for the effective degradation of BPA [24]. First, positively charged NMS-cetrimonium bromide was synthesized, followed by the addition of sodium sulfide nonahydrate and 0.5 wt% cetrimonium bromide aqueous solution. Consequently, equal weights of NMS-cetrimonium bromide and delaminated negatively charged MXene layers were integrated to obtain NMS@MX. The BPA removal rates of 38.1 %, 61.3 %, and 91.6 % by nickel magnesium oxide, nickel magnesium sulfide, and NMS@MX, respectively, indicated the superior catalytic performance of NMS@MX. As shown in Fig. 5b, the BPA degradation kinetics conformed to a pseudo-first-order reaction. Increasing the initial BPA concentration in 10 mg L^−1^ intervals weakened the sonophotocatalytic ability, likely because of the poor availability of catalyst active sites; at 10 mg L^−1^, the degradation rate (ln C_o_/C_t_) was approximately three times greater than that at 50 mg L^−1^. The degradation efficiencies of the combined processes are shown in Fig. 5c. To test the degradation efficiency of sonication and visible light, a 10 mg L^−1^ BPA solution was treated with 1,000 mg L^−1^ catalyst under 970 kHz and 400 W ultrasound. Although sonication or visible light alone resulted in negligible mineralization, the NMS@MX + visible light system improved the degradation efficiency to approximately 60 %, which further increased to 90 % upon sonication. In the combined system, NMS@MX was likely activated in the visible light regions of 270–700 nm, because the low bandgap of MXene (1.6 eV) effectively narrowed the higher bandgap of NMS (2.97 eV). According to its relatively negative reduction potential, the covalent band of MXene could reduce O_2_ into O_2_^•-^, which was subsequently converted into ^•^OH. These radicals, in conjunction with the photogenerated holes on MXene, induced the effective mineralization of BPA [134].

#### 2.2.2 Carbon material-based catalysts

According to its estimated bandgap (2.7 eV), GCN was successfully activated under visible light ranging from 400 to 420 nm in a 10 mg L^−1^ BPA solution [36]. The reaction constant increased from 2.0 × 10^-3^ min^−1^ at a catalyst dose of 0.01 g L^−1^ to 14.0 × 10^-3^ min^−1^ with a catalyst dose of 0.1 g L^−1^, exhibiting synergy with ultrasonication. Furthermore, the observed decrease in H_2_O_2_ consumption following GCN incorporation was presumably due to the direct degradation of BPA within the holes of the catalyst [36]. In a separate experiment, SnS_2_/CNFs catalysts were synthesized through a solvothermal method involving the uniform growth of 0.5 mol SnS_2_ nanosheets on a 2 × 4 cm^2^ CNF membrane [35]. Sonocatalytic degradation with the catalyst achieved nearly 100 % removal of both Cr(VI) and BPA, with rate constants of 0.132 and 0.04 min^−1^, respectively. This high efficiency was attributed to the rapid electron transport enabled by the conductivity of the CNF membranes and the intrinsic electric field of SnS_2_, which enhanced electron-hole separation. Specifically, Cr(VI) was reduced by thermally excited e^-^, whereas BPA was oxidized by ^•^OH radicals generated from H_2_O_2_. Furthermore, degradation efficiency increased with ultrasonic power, thereby confirming the contribution of piezoelectric effect induced by the mechanical distortion of SnS_2_ [35].

The synergistic effect between persulfate and sludge biochar has been previously reported [85]. Modified with FeSO_4_·7H_2_O, 2 g L^−1^ sludge biochar was applied to 20 mg L^−1^ BPA solution. The experiments were conducted with 3 mM persulfate under 60 W sonication. Performance comparison revealed limited BPA removal with ultrasonication only (nearly 20 %; k = 2.8 × 10^-3^ min^−1^) and BC/ultrasonication (27.2 %; k = 5.09 × 10^-3^ min^−1^), whereas the BC/ultrasonication/persulfate system demonstrated 97.8 % removal (k = 3.73 × 10^-2^ min^−1^). This enhancement was attributed to the efficient conversion of persulfate into SO_4_^•-^ and ^•^OH by the surface Fe^2+^ on the catalyst. These radicals were simultaneously generated from ultrasonication-facilitated thermal decomposition of persulfate and H_2_O [135]. Salova et al. synthesized a LaFeO_3_/methylcellulose/multi-walled carbon nanotubes-NiCu_2_O_4_/Zn catalyst through a co-precipitation method assisted by microwave irradiation [37]. The catalyst (0.5 g L^−1^) was applied to 3 mg L^−1^ BPA solution under ultrasonic conditions of 70 W and 40 kHz, achieving 91.2 % degradation (k = 0.078 min^−1^). The effectiveness of the sonocatalytic process was attributed to the augmentation of ultrasonication waves and the photocatalytic ability of the nanocomposite. First, NiCu_2_O_4_ nanoparticles could attenuate the speed of sound, produce microbubbles, or create local spots of intense sound, promoting the generation of reactive species [136]. Additionally, sonoluminescence caused by the absorption of ultrasonication waves could facilitate photoexcitation of electrons, thereby inducing photocatalytic effects [137].

### 2.3 Degradation affected by hybrid processes involving O_3_, Fenton, or UV

**Fig. 6:**
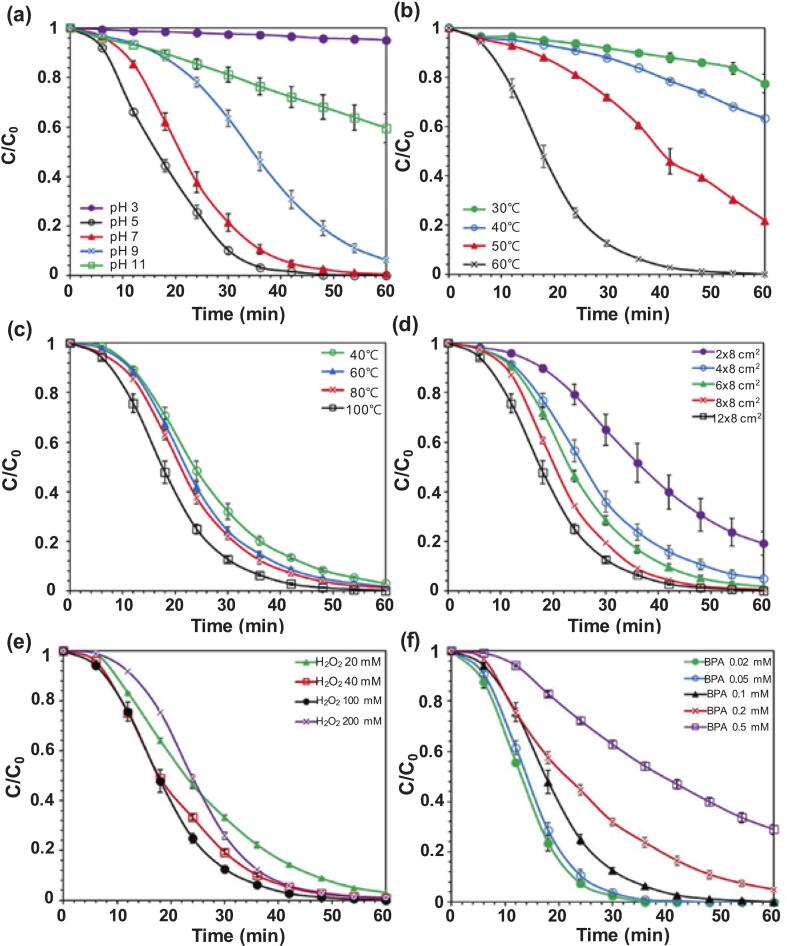
Effect of (a) solution pH, (b) temperature, (c) ultrasonic power, (d) CF size, (e) initial H_2_O_2_ concentration, and (f) initial BPA concentration on BPA degradation in sono-Fenton-like process ([BPA] = 0.1 mM, CF size = 12 × 8 cm, [H_2_O_2_] = 100 mM, temperature = 60 °C, US power = 96.57 W) [83].

Combining ultrasonication with AOPs (e.g., O_3_, Fenton reaction, and UV irradiation) significantly improves the degradation of emerging contaminants owing to highly reactive radicals (^•^OH, O_2_^•-^, and O_3_) and cavitation effects, leading to enhanced removal efficiency, while degradation in these hybrid systems is also affected by direct ozonation, ferric iron reduction, and photolysis [31]. Various pH levels were utilized in the ultrasonication-Fenton hybrid system to examine the effects of CF on BPA degradation [83]. Complete removal was achieved at pH 5 and 7, whereas approximately 90 % and 40 % removal efficiencies were achieved at pH 9 and 11, respectively (Fig. 6a). In acidic or neutral solutions, sufficient H^+^ can enhance ^•^OH generation by accelerating the oxidation of Cu^+^ to Cu^2+^ [138]. However, at pH 3 and 11, H^+^ and OH^–^ could consume ^•^OH to form water and oxygen, thereby reducing the degradation efficiency. As shown in Fig. 6b, the increase in the removal rates at higher temperatures was possibly due to the endothermic nature of copper reduction and ionization, as well as the exponential dependence of copper oxidation on the Arrhenius law [139]. Fig. 6c shows that increasing the ultrasonication power from 38.6 to 96.6 W (40 % to 100 % sonicator output) enhanced contaminant removal, due to the intensified mixing caused by the turbulence from bubble collapse [113]. As shown in Fig. 6d, removal rates rose from 80.9 % ± 4.74 % (0.12 g; 2 × 8 cm^2^) to nearly 100 % (0.70 g; 12 × 8 cm^2^), because of the accumulation of ^•^OH which could promote greater utilization of catalytic active sites [101]. The relatively slow reaction speed with 20 mM H_2_O_2_ was attributed to its stoichiometric depletion, which allowed O_2_ to suppress the ^•^ OH-generation reaction between Cu^+^ and H_2_O_2_ (Fig. 6e) [140]. Finally, increasing the initial BPA concentration lowered the reaction speed, as the competitive occupation of the catalyst surface by the contaminant supposedly suppressed the ionization of copper [141]. However, the likelihood of collisions between BPA and short-lived ^•^OH could also increase, enhancing overall contaminant removal [138]. Once the BPA concentrations were 0.10, 0.20, and 0.50 mM, 0.10, 0.19, and 0.36 mM of BPA was degraded, respectively, as shown in Fig. 6f [83].

H_2_O_2_, Fe^2+^, UV irradiation, and the Fenton reaction were applied in conjunction with sonolysis in a 10 mg L^−1^ BPA solution under sonication conditions of 40 kHz and 200 W [38]. The addition of H_2_O_2_ had a relatively low effect on total BPA degradation which was measured at 19.9 % ± 0.67 % and 20.0 % ± 0.71 % for sonolysis and sonolysis/H_2_O_2_, respectively. The radical scavenging of H_2_O_2_ may have contributed to the low utilization of ^•^OH. In the sonolysis/Fe^2+^ system, the enhancement to 33.9 % ± 1.87 % degradation was attributed to the improvement in ^•^OH regeneration, demonstrating the dominance of the Fenton reaction over sonolysis regarding ^•^OH production. The highest reaction rate of sono-Fenton reactions was observed under aerated conditions at atmospheric static pressure (9.22 × 10^-3^ s^−1^), whereas de-aeration and a rise in static pressure resulted in 4.98 × 10^-3^ s^−1^ and 3.67 × 10^-3^ s^−1^, respectively. Dissolved oxygen could promote BPA oxidation by conserving the radicals and contributing to the generation of additional HO_2_^•^ [142]. In addition, the transient cavitation induced by sonication was assumed to cause intense convection, leading to relatively high interaction rates between the radicals and BPA at atmospheric static pressure. Finally, UV irradiation with a peak emission of 365 nm was combined with the sono-Fenton reaction, resulting in a kinetic constant of 2.6 × 10^-3^ s^−1^ which was slower than that of sono-Fenton alone (9.22 × 10^-3^ s^−1^). This negative synergy was attributed to the inefficient utilization of H_2_O_2_, as the UV irradiation pathway competed with the Fenton reaction, which is the dominant source of ^•^OH. Moreover, it was assumed that the insufficient emission intensity at the H_2_O_2_ absorption peak (254 nm) contributed to ineffective degradation [38].

Manganese dioxide nanoparticles (α-MnO_2_/BCs) were hydrothermally integrated onto BC nanocomposite surfaces to evaluate sonocatalytic degradation of BPA with H_2_O_2_ [143]. Over 120 min, ultrasonication alone achieved only 15 % degradation, whereas the ultrasonication/H_2_O_2_ hybrid system exhibited a significant improvement of 75 % degradation. Moreover, the addition of α-MnO_2_/BCs to the hybrid system led to nearly complete BPA degradation within 20 min. The synergy factor between ultrasonication and the catalyst was approximately 2.7, implying efficient H_2_O_2_ decomposition. These effects were explained by improved production of ^•^OH and O_2_^•-^ [144]. Additionally, HO_2_^•^ was generated through reactions between α-MnO_2_/BCs and H_2_O_2_, enabling the oxidation of lattice oxygen [145]. In a separate photo-Fenton experiment, the effect of varying H_2_O_2_ concentration on BPA degradation was investigated [62]. Among the tested conditions, 4.8 mM H_2_O_2_ achieved the highest degradation rate of 36.6 % and total organic carbon reduction of 8.0 %. However, at higher stoichiometric ratios, H_2_O_2_ may have scavenged ^•^OH via ^•^OH recombination reactions [62]. Comparable improvements were observed in a hybrid process involving 10 mg L^−1^ O_3_, where BPA degradation increased by approximately 15 % [44]. This effect was ascribed to the attack by ^•^OH or the molecular O_3_ itself. ^•^OH was likely produced through decomposition of O_3_. Meanwhile, given its oxidation potential of 2.07 V, which exceeds that of O_2_ or H_2_O_2_, O_3_could directly oxidize BPA by attacking the carbon double bonds connecting the aromatic rings [44].

A perovskite catalyst was assembled using the sol–gel technique in a sonophoto-Fenton system [146]. Ultrasonication alone achieved 13.5 % degradation, whereas degradation efficiency increased to 19.9 % upon the addition of H_2_O_2_ and perovskite. When combined with visible light, the ultrasonication/perovskite/H_2_O_2_ system exhibited a marginal increase in degradation to 21.8 %. This suggested that the catalytic activity of perovskite was more effectively triggered by ultrasonication than by visible light. Specifically, ultrasonication could create hot spots on the surface of the perovskite, generating ^•^OH via H_2_O pyrolysis [147]. Simultaneously, sonoluminescent light with wavelengths below 375 nm may have promoted photocatalytic effects [148]. Table 1 summarizes previous research on the degradation of BPA using various catalysts and hybrid processes.

**Table 1:** Summary of BPA during sonocatalytic treatment.

C_0_ (mg L^−1^)	Catalysts orhybrids	Experimental conditions	Key removal orrate constant	Ref.
10	UV Fe^2+^PDS	SDW 1.7 MHz 25 W	65 % (US + Fe^2+^/US + PDS, 60 min) 97/> 99 % (US + UV + Fe^2+^+PDS, 60/90 min)	[45]
10	HC Fe^2+^PDS	SDW 1.7 MHz 240 min	4.23 × 10^-4^ min^−1^ (US + HC) 5.4 × 10^-4^ min^−1^ (US + HC + PDS) 97 % (US + HC + PDS + Fe^2+^)	[49]
0.1	PMSrGO	SDW 35 kHz 240 W	> 99 % (US + rGO + PMS)	[34]
20	RB-MBC RH-MBC H_2_O_2_	SDW 51.95 W mL^−1^ 40 min	10.5 % (RB-MBC) 13.5 % (RB-MBC + H_2_O_2_) 94.3 % (US + RB-MBC + H_2_O_2_)	[33]
20	CF H_2_O_2_	SDW 37 kHz 96.57 W 60 min	23.0 % (CF + H_2_O_2_) 100 % (US + CF + H_2_O_2_)	[83]
10	H_2_O_2_ Fe^2+^	SDW 40 kHz 8.71 W L^−1^ 10 min	7.66 × 10^-5^ min^−1^ (US) 1.09 × 10^-4^/3.18 × 10^-4^ min^−1^ (US + H_2_O_2_/US + Fe^2+^) 3.2 × 10^-3^ min^−1^ (US + H_2_O_2_ + Fe^2+^) 2.6 × 10^-3^ min^−1^ (US + H_2_O_2_ + Fe^2+^+UV)	[38]
20	PDSCF	SDW 37 kHz 97.17 W 30 min	1.44 % (US) 89.7 % (US + PDS) 99.5 % (US + PDS/US + CF)	[23]
1	O_3_	SDW/NSW 20 kHz 57 W 30 min	71/75 % (US, SDW/NSW) 92 % (US + O_3_, SDW)	[44]
1	UV	WWE 20 kHz 43 W cm^−2^ 30 min	92 % (US) 95 % (US + UV)	[43]
0.45	PDS	SDW 35 kHz 20 W L^−1^ 150/180 min	86 % (US, 180 min) > 99 % (US + PDS, 150 min)	[66]
10	HNPsPDS	SDW 250 W 15 min	35.9 % (US) 75.4 % (US + PDS) 84.6 % (US + HNPs) 98.9 % (US + PDS + HNPs)	[80]
20	BCPS	UPW 60 W 80 min	20 % (US) 27.2/33.6 % (US + BC/US + PS) 98 % (US + BC + PS)	[85]
1	PDSEC	SDW 24 kHz 25/125 μm 30 min	38/61 % (US, 25/125 μm) 93 % (US + PDS + EC, 125 μm)	[111]
15	H_2_O_2_ LaFeO_3_Vis	SDW 20 kHz 40 W 180 min	13.5 % (US) 19.8 % (US + H_2_O_2_ + Vis) 19.9 % (US + H_2_O_2_ + LaFeO_3_) 21.8 % (US + H_2_O_2_ + LaFeO_3_ + Vis)	[146]
15	H_2_O_2_ LaFeO_3_Vis	SDW 20 kHz 40 W 180/360 min	36.6 % (US + H_2_O_2_ + Vis + 0.5 g L^−1^ LaFeO_3_, 180 min) 57.8 % (US + H_2_O_2_ + Vis + 0.75 g L^−1^ LaFeO_3_, 360 min)	[62]
12.5	CoFeO-GO CoFeO-GCN PSVis	SDW 40 kHz 84 W 45/80/90 min	34 % (US + PS + CoFeO-GCN, 90 min) 41 % (US + PS + CoFeO-GO, 90 min) 100 % (US + PS + Vis + CoFe_2_O_4_-gC_3_N_4_/CoFe_2_O_4_-GO, 45/80 min)	[39]
0.5	Pd CeO_2_	UPW 20 kHz 71/96 W L^−1^ 60 min	0.0240 min^−1^ (US) 0.0211 min^−1^ (US + CeO_2_) 0.0516 min^−1^ (US + CeO_2_ + Pd)	[57]
25	NaCl ZnO Na_2_SO_4_ CCl_4_ H_2_O_2_	SDW 40 kHz 120 W 25 min	28 % (US) 44/48/52/70/84 % (US + NaCl/US + ZnO/US + Na_2_SO_4_/US + CCl_4_/US + H_2_O_2_)	[157]
10	Ag_3_PO_4_-Fe_3_O_4_@AB BC AB Fe_3_O_4_@AB Fe_3_O_4_	SDW 970 kHz 177 W L^−1^ 60 min	38 % (US) 46/50/58/68/82 % (US + Fe_3_O_4_/US + Fe_3_O_4_@AB/US + AB/US + BC/US + Ag_3_PO_4_-Fe_3_O_4_@AB)	[131]
20	PBC α- MnO_2_/BCs δ-MnO_2_/BCs	SDW 20 kHz 130 W 120 min	15 % (US) 75 % (US + H_2_O_2_) 100 % (US + PBC + H_2_O_2_ + δ-MnO_2_/BCs/US + H_2_O_2_ + α-MnO_2_/BCs)	[143]
0. 01 μg L^−1^	T-MNSABF	SDW 72 kHz 375 min	9 % (US) 100 % (US + BF)	[158]
10	hBBNP β-Bi_2_O_3_	SDW 0/35/72/100/170 kHz 0.5 W cm^−2^ 360 min	34 % (US, 100 kHz) 71/100 % (US + hBBNP, 100/35 kHz) 59/100 % (US + β-Bi_2_O_3,_ 100/35 kHz)	[102]
40	Sch H_2_O_2_	SDW 20 kHz 60 min	6.1 % (US) 6.8 % (US + Sch) 9.5 % (US + H_2_O_2_) 98.0 % (US + Sch + H_2_O_2_)	[71]
40	CuS/BaWO_4_ BaWO_4_	SDW 40 kHz 500 W 60 min	20 % (US) 49.7 % (US/BaWO_4_) 71.0 % (US + CuS/BaWO_4)_	[98]
10	Sr_x_Ba_1-x_-TiO_3_ (0.7 ≤ x ≤ 1.0)	SDW 40 kHz 300/240/180 W 16 min	0.239/0.145/0.115 min^−1^; 99/90/86 % (US + Sr_x_Ba_1-x_-TiO_3_) (300/240/180 W)	[130]
10	α-MnO_2_PDS	WWE/DDW 37 kHz 70 W	88.8/> 99 % (US + α-MnO_2_ + PDS_,_ WWE/DDW)	[86]
25	CCl_4_ FeSO_4_EAC	SDW 1146/864/580 kHz 50 W 25/60 min	7/39/38 % (US, 1146/864/580 kHz) 70 % (US + CCl_4_, 25 min) 72 % (US + FeSO_4_/US + EAC, 60 min)	[153]
0.02	TiO_2_UV	SDW 20 kHz	1.349 min^−1^ (US + TiO_2_ + UV)	[122]
4	PS UV Fe^2+^	SDW/NSW 1.7 MHz 3 W cm^−2^ 40 min	7 % (US, SDW/NSW) > 99/93 % (US + UV + PS + Fe^2+^, SDW/NSW)	[159]
5	TiO_2_ Er: Y_2_O_3_ Er: Y_2_O_3_@SiO_2_ Er: Y_2_O_3_ @SiO_2_@mTiO_2_	SDW 300 kHz 30 min	0.054/0.077/0.086/0.096/0.155 min^−1^ (US + TiO_2_/US + Er: Y_2_O_3_/US + Er: Y_2_O_3_@SiO_2_ /US + Er: Y_2_O_3_@SiO@mTiO_2_)	[125]
10	TiO_2_ Fe_3_O_4_@mTiO_2_ Fe_3_O_4_@mTiO_2_@ mc/SiO_2_	SDW 300 kHz 1000 W L^−1^ 60 min	80 % (US) 90 % (US + TiO_2_) 100 % (US + Fe_3_O_4_@mTiO_2_, US + Fe_3_O_4_@mTiO_2_@mc/SiO_2_)	[160]
1.0–0.1	UV TiO_2_	SDW/WWE 300 kHz 120 min	100 % (US + UV + TiO_2_, SDW/WWE) 3.0 × 10^-2^/4.4 × 10^-2^ min^−1^ (US + UV + TiO_2_, SDW/WWE)	[123]
15	LFO/MC/MWCNT LFO/MC/MWCNT-NCOLFO/MC/MWCNT-NCO/Z	SDW 40 kHz 70 W 30 min	0.033 min^−1^ (US + LFO/MC/MWCNT) 0.053 min^−1^ (US + LFO/MC/MWCNT-NCO) 0.078 min^−1^ (US + LFO/MC/MWCNT-NCO/Z)	[37]
10	CoTiO_3_ Ti_3_C_2_T_x_CoT@MX	SDW/NSW/WWE 970 kHz 300 W 90 min	38.4/46.3/96.9 % (US + CoTiO_3_/US + TiC_2_T_x_/ US + CoT@MX, SDW) 81.1/89.9 % (US + CoT@MX, NSW/WWE)	[32]
8.56	HSO_5_^-^ H_2_O_2_ S_2_O_8_^2-^ IO_4_^-^	SDW 35 kHz 116 W L^−1^	7.2 × 10^-3^/7.5 × 10^-3^/11.3 × 10^-3^/16.3 × 10^-3^ min^−1^ (US + HSO_5_^-^/US + H_2_O_2_/US + S_2_O_8_^2-^/US + IO_4_)	[20]
10	GCNVis	SWW 35 kHz 50 W 120 min	74 % (US) 80.6 % (US + GCN + Vis)	[36]
10	TiO_2_UV	SWW 35 kHz 50 W	22 % (US) 60/78 % (US + TiO_2_/US + UV) 96 % (US + TiO_2_ + UV)	[63]
30	MNPC PMSUV	SDW 200 W 30 min	15.7 % (US) 59.7 % (US + PMS + UV) 73.2 % (US + UV + MNPC) 83.3 % (US + PMS + MNPC) > 99 % (US + PMS + UV + MNPC)	[149]
10	SnS_2_ CNFs SnS_2_/CNFs	SDW 40 kHz 300 W 120 min	10 % (US + CNFs) 42 % (US + SnS_2_) 100 % (US + SnS_2_/CNFs)	[35]
10	NMS@MXeneVis	SDW 970 kHz 400 W 720 min	48 % (US) 75 % (US + NMS@MXene) 91 % (US + NMS@MXene + Vis)	[24]
2	PMS	SDW 20/200/400 kHz 90/110/140 W L^−1^ 30 min	0.04/0.051/0.063 min^−1^ (US) 0.051/0.085/0.140 min^−1^ (US + PMS) (20/200/400 kHz)	[107]
10	BiOI BiOI/ZnO NRs ZnO NRsVis	SWW 40 kHz 90 W 30/120 min	75 % (US + BiOI, 120 min) 90 % (US + ZnO NRs, 120 min) 100 % (US + BiOI/ZnO NRs + Vis, 30 min)	[161]
10	STOV-STO NFs	SDW 40 kHz 60/90/120/150 W L^−1^ 24 min	47 % (US + STO, 150 W) 42/85/90/100 % (US + V-STO NFs) (60/90/120/150 W)	[162]
10	Bio-MOF-1PMS	SDW/WWE 40 kHz 100 W 40 min	16.0 % (US, SDW) 29.0/69.0 % (US + PMS/US + Bio-MOF-1, SDW) 67.7/86.7/95.4/98.5 % (US + PMS + Bio-MOF-1, WWE-1/WWE-2/WWE-3/SDW)	[53]

α- MnO_2_/BCs = urchin-like manganese dioxide biochar nano composites; AB = activated biochar; Ag_3_PO_4_-Fe_3_O_4_@AB = Ag_3_PO_4_-Fe_3_O_4_ on activated biochar; δ-MnO_2_/BCs = flower-like manganese dioxide biochar nanocomposites; BC = biochar; BF = bismuth ferrite; BiOI/ZnO NRs = BiOI/ZNO nano rods; Bio-MOF-1 = biological metal organic framework-1; C_0_ = BPA initial concentration; CF = waste antivirus copper film; CNFs = carbon nanofibers; CoFeO-GO = CoFe_2_O_4_ graphene oxide; CoFeO-GCN = CoFe_2_O_4_ graphitic carbon nitride; CoT@MX = CoTiO_3_@Ti_3_C_2_T_x_; DDW = deuterium depleted water; EAC = ethyl anthraquinone; EC = electrochemical degradation; Er: Y_2_O_3_ = erbium doped yttrium oxide; Er: Y_2_O_3_@SiO_2_ = erbium doped yttrium core with silica layer; Er: Y_2_O_3_@SiO_2_@mTiO_2_ = core–shell Er: Y_2_O_3_@SiO_2_ nanospheres on mesoporous TiO_2_; Fe_3_O_4_@AB = Fe_3_O_4_ on activated biochar; Fe_3_O_4_@mTiO_2_ = core shell structured Fe_3_O_4_ on mesoporous TiO_2_ nanospheres; Fe_3_O_4_@mTiO_2_@mc/SiO_2_ = mesoporous C/SiO_2_ framework wrapped magnetic mesoporous TiO_2_ composites; GCN = graphitic carbon nitride; GO = graphene oxide; hBBNP = hetero structured β-Bi_2_O_3_/Bi_2_O_2_CO_3_ nanoplates; HC = hydrodynamic cavitation; HNPs = hematite nanoparticles; LFO/MC/MWCNT = LaFeO_3_ methyl cellulose multi-walled carbon nanotubes; LFO/MC/MWCNT-NCO = LaFeO_3_ methyl cellulose multi-walled carbon nanotubes-NiCu_2_O_4_; LFO/MCWCNT-NCO/Z = LaFeO_3_ methyl cellulose multi-walled carbon nanotubes-NiCu_2_O_4_/Zn nano composite; MNPC = magnetite nanoparticles supported on carbon; NMS@MXene = Ni_x_Mg_4-x_S_4_ on MXene; NSW = natural surface water; PBC = pristine biochar; PMS = peroxymonosulfate; PS = persulfate; rGO = reduced graphene oxide; RB-MBC = rice bran magnetic biochar; RH-MBC = rice husk magnetic biochar; Sch = Schwertmannite (Fe_8_O_8_(OH)_8-2x_(SO_4_)_x,_ 1 ≤ x ≤ 1.75); SDW = synthetic drinking water; SnS_2_/CNFs = SnS_2_ carbon nanofibers; STO = SrTiO_3_; V-STO NFs = V-doped SrTiO_3_ nanofibers; SWW = synthetic wastewater; T-MNSA = tris coated magnetic nano structured adsorbent; UPW = ultra-pure water; US = ultrasonication; UV = ultraviolet; Vis = visible light; WWE = wastewater effluent; ZnO NRs = ZnO nanorods.

### 2.4 Mineralization pathways of BPA

**Fig. 7:**
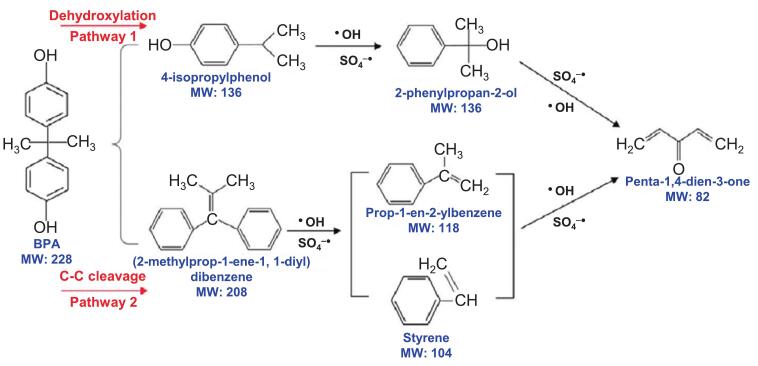
Proposed pathway schemes for degradation of BPA by MNPC + UV + US + PMS process [149].

The evaluation of BPA mineralization during sonocatalytic degradation is significant for assessing the overall efficiency and effectiveness of the treatment process. A magnetite heterogeneous catalyst supported on carbon (MNPC) consisting of 58 % carbon, 28 % oxygen, and 14 % iron was integrated under alkaline conditions via the chemical co-precipitation of ferrous and ferric ions [149]. In separate systems, magnetite nanoparticles, powdered activated carbon, and MNPC alone removed 8.30 %, 22.1 %, and 26.4 % of BPA, respectively, mainly by adsorption. The implementation of ultrasound and UV irradiation in an identical process resulted in the complete removal of BPA. This improvement was attributed to the concurrent generation of ^•^OH and SO_4_^•-^. In this system, two primary mineralization pathways of BPA were identified (Fig. 7). In the first pathway, cleavage of the C–C bond converted BPA into 4-isopropylphenol. Weakening of the bonds was attributed to the increased electron density in the phenol groups, which was resulted by the electron-donating effect of the hydroxyl groups. The generated radicals, ^•^OH and SO_4_^•-^, further decomposed 4-isopropylphenol into 2-phenylpropan-2-ol and eventually into penta-1,4-dien-3-one. In the second pathway, BPA underwent dehydroxylation to producing dibenzene, which dissociated into prop-1-en-2-ylbenzene and styrene. The reactive radicals then deprotonated these compounds, ultimately generating penta-1,4-dien-3-one through ring-opening. These findings elucidated the crucial role of oxidizing agents in BPA degradation. In addition, the inhibition of oxygen consumption decreased from 26.7 % to 0.04 % after 60 min, indicating a reduction in toxicity [149].

Consistent with previous findings [150], mass spectrometry confirmed formation of several BPA degradation by-products, including 4-[2-(4-hydroxyphenyl) propan-2 yl] benzene-1,2-diol, 4,4′-isopropylidene bis catechol, 4′-hydroxyacetophenone, and 4-hydroxybenzoic acid [43]. When sonication and UV irradiation were combined, these intermediates appeared earlier in the reaction along with additional compounds such as 4-(2 hydroxy-2-propanyl) phenol and 1,4-benzoquinone. These oxidized intermediates, primarily formed via the hydroxylation of BPA, contribute to the toxicity of the treated solution, ranging between 8 % and 60 % [151].

Mineralization pathway studies in the ultrasonication + IO_4_^-^ process revealed the preeminent generation of 1,3-diphenylpropanetriide with double bonds, suggesting that the hydroxyl and methyl groups of BPA were initially attacked [20]. Another possible pathway was the production of BPA free radicals through aromatic ring attack, followed by β-scission and formation of phenol, 4-isopropenylphenol, or 4-hydroxyacetophenone [152]. Mineralization of BPA was observed in another degradation experiment that accompanied CCl_4_, FeSO_4_·7H_2_O, and ethyl anthraquinone [153]. The mineralization rates for the optimal parameters were 35 %, 24 %, and 32 %, respectively. More precisely, the presence of CCl_4_ accelerated mineralization and accounted for species such as 4-hydroxyphenacyl alcohol, 4-prophylbenzyl acetaldehyde, and p-phenyl-p-benzyl-isopropane, which is suggested to be highly toxic. The same compounds were formed in the case of ethyl anthraquinone and FeSO_4_·7H_2_O, with the latter producing a higher number of byproducts during the first 15 min [153].

According to the degradation pathway studies of BPA and MB, both contaminants produced ultimate products such as CO_2_ and H_2_O [86]. First, the intermediates of BPA are mainly generated through C–C cleavage, bridge cleavage, hydrogen deprivation from the BPA molecule, and aromatic ring opening. Oxalic acid, 2-methylpentyl alcohol, and adipic acid were generated by radical-influenced ring cleavage, as previously reported [154]. In addition, the by-products of MB, such as N, N-dimethylaniline and p-dimethylaminoaniline, are generally formed by thiazide ring-opening followed by the destruction of carbon–nitrogen bonds [155]. The degradation pathway of BPA utilizing GCN was investigated via extraction ion chromatography for 300 min under 35 kHz ultrasound [36]. Based on the peak area versus retention time, the initial attack by ^•^OH on BPA converted it into mono-and dihydroxylated BPA, which was consistent with other reports [156]. Monohydroxylated BPA was likely converted to 4 hydroxyacetophenone, whereas dihydroxylated BPA was degraded to 4 hydroxybenzaldehyde and 4-isopropenylphenol. Although 4-isopropenylphenol may have subsequently broken down into CO_2_ and H_2_O, 4-hydroxyacetophenone and 4 hydroxybenzaldehyde persisted for 300 min [36].

Mineralization studies have demonstrated the dominance of mono-hydroxylated and tri-hydroxylated BPA over the first 2 and 4 h, respectively [66]. Concurrently, the majority of the oxidized compounds reached their peak concentrations within the same time frame, indicating the simultaneous occurrence of hydroxylation and oxidation. The hydroxylated derivatives are likely formed via the electrophilic attack of ^•^OH on the aromatic rings [150]. Despite the moderate acute toxicity of 42 % observed at 5 mg L^−1^ BPA, the toxic effects declined as the reaction progressed, indicating the formation of less toxic intermediates [66].

## 3 Areas for future study

Despite the substantial progress described in this review, future investigations remain essential in several areas of BPA sonocatalysis. A significant constraint is the limited demonstration of sustained performance under realistic conditions. In most studies, water matrices have been simplified, limiting their applicability to complex natural and wastewater matrices. Quantitative analysis is required to understand the influence of competing contaminants and fluctuating operational conditions on radical production and reaction pathways. Future investigations should also focus on developing optimized reactors designed to mitigate acoustic field attenuation when applied to large-scale continuous operations. There is a lack of understanding of the catalyst surface configuration and the role of certain reactive oxygen species. The development of molecular simulation models specific to sonocatalysis is crucial for designing catalysts with optimal electronic and surface properties for enhanced catalytic activity. Concurrently, the precise quantification of other reactive oxygen species (e.g., ^1^O_2_, O_2_^•-^, and HO_2_^•^) and their respective contributions to BPA degradation requires active investigation. A knowledge gap lies in the characterization of degradation intermediates and their toxicological profiles. Although some studies have identified specific by-products, a comprehensive assessment of their persistence, bioaccumulation potential, and ecological impact is lacking. Given that certain intermediates may pose comparable or greater toxicity than BPA itself, future investigations should integrate advanced analytical methods with ecotoxicological assays to prevent the inadvertent production of harmful transformation products. Catalyst development faces fundamental challenges related to active site poisoning and metal ion leaching. Therefore, research on effective catalyst regeneration strategies is required to prevent catalyst loss and secondary contamination. Finally, a major economic barrier to ultrasound treatment is high energy consumption. Future studies should focus on process intensification through the development of synergistic hybrid systems, including the integration with existing water and wastewater treatment processes such as coagulation/flocculation, activated sludge, and chlorination. Moreover, there is a need for innovation in sonochemical reactor design to optimize acoustic energy distribution, maximize active cavitation volume, and enhance mass transport phenomena, thereby reducing energy requirements and improving economic efficiency.

## 4 Conclusions

The global presence of BPA in water systems necessitates the development of effective remediation strategies. Therefore, this review examines the current state of knowledge regarding sonocatalytic removal of BPA, the efficacy of which is based on the integration of acoustic waves with catalyst chemistry. Through ultrasonic cavitation, the violent collapse of the microbubbles created localized hotspots that generated highly reactive ^•^OH and SO_4_^•-^ for BPA degradation. The influences of solution chemistry, operational parameters, and contaminant characteristics were elucidated. The solution pH was identified as a strongly relevant factor, with optimal degradation typically occurring within pH 5–7 where BPA maintained its neutral form. Elevated temperatures led to enhanced reaction rates, but could compromise the bubble collapse intensity, demanding careful optimization. The presence of background ions and natural organic matter generally inhibited BPA degradation by scavenging radicals or forming less reactive oxidants, although certain ions acted as promoters. Degradation performance was suppressed by scavengers and organic surfactants, indicating the involvement of O_2_^•-^ and ^1^O_2_ in BPA degradation.

**Fig. 8:**
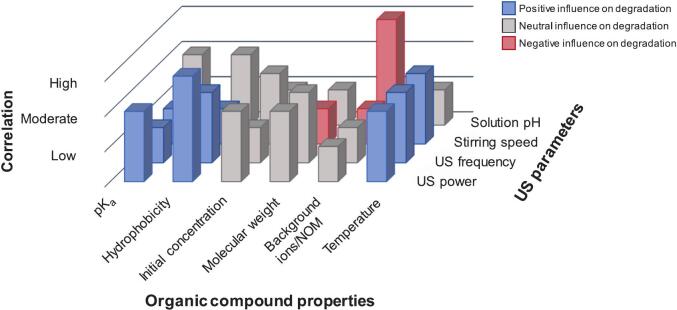
Anticipated correlation between US parameters and BPA/organic compound properties and their impact on BPA/organic compound degradation based on data presented in Table 1. Correlation indicates the significance of combined effects of BPA/individual organic compound properties and US.

Operational settings, including frequency, power, and mode, directly influenced cavitation dynamics. Lower frequencies caused bubble collapse, whereas higher frequencies increased the overall ^•^OH production. In general, optimal power levels improved the degradation efficiency, whereas excessive intensity resulted in cavitation instability. In recent years, nanostructured and hybrid catalysts, such as MXenes, CNFs, and heterojunctions, have been increasingly used. These materials exhibited improved active site exposure, electron-hole separation, and light absorption, leading to faster reactions and higher mineralization rates. Hybrid systems incorporating ozone, UV, or Fenton chemistry further stimulated radical production, although the scavenging effect of H2O2 was stimulated to some extent. Mineralization pathway studies confirmed the persistence of intermediates, such as hydroxyacetophenone and hydroxybenzaldehyde, emphasizing the importance of monitoring the varying toxicities of the by-products. Overall, the integration of sonocatalysis and hybrid oxidation for BPA removal is a technologically viable approach. Over a decade of research has consistently demonstrated removal efficiencies exceeding 90 % under optimized conditions across diverse experimental processes. However, a more thorough understanding of the underlying mechanisms is required for the transition from empirical lab-scale procedures to predictable large-scale processes. Fig. 8 illustrates the projected correlation between ultrasonication parameters and the properties of BPA and other organic compounds, highlighting their influence on the degradation of BPA and similar organic compounds. This correlation is derived from this in-depth review, providing insights into how these factors interact to affect degradation processes.

## CRediT authorship contribution statement

**Hyunjin Shin:** Writing – original draft, Conceptualization. **Hak-Hyeon Kim:** Writing – original draft, Conceptualization. **Sujin An:** Resources, Formal analysis. **Narae Yang:** Methodology, Data curation. **Chang Min Park:** Methodology, Formal analysis. **Min Jang:** Validation, Methodology. **Byung-Moon Jun:** Writing – review & editing, Validation, Supervision, Resources. **Yeomin Yoon:** Writing – review & editing, Validation, Supervision.

## Declaration of competing interest

The authors declare that they have no known competing financial interests or personal relationships that could have appeared to influence the work reported in this paper.
